# Predicting Acute Postoperative Pain Trajectories and Long-Term Outcomes of Adolescents after Spinal Fusion Surgery

**DOI:** 10.1155/2020/9874739

**Published:** 2020-02-24

**Authors:** Don Daniel Ocay, Mandy M. J. Li, Pablo Ingelmo, Jean A. Ouellet, M. Gabrielle Pagé, Catherine E. Ferland

**Affiliations:** ^1^Department of Experimental Surgery, McGill University, Montreal, QC, Canada; ^2^Shriners Hospitals for Children-Canada, Montreal, QC, Canada; ^3^Faculty of Medicine, McGill University, Montreal, QC, Canada; ^4^Chronic Pain Services, Montreal Children's Hospital, Montreal, QC, Canada; ^5^Department of Anesthesia, McGill University, Montreal, QC, Canada; ^6^Department of Pediatric Orthopedics, McGill University, Montreal, QC, Canada; ^7^Department of Anesthesiology and Pain Medicine, Université de Montréal, Montreal, QC, Canada; ^8^Centre de Recherche Du Centre Hospitalier de L'Université de Montréal, Montreal, QC, Canada; ^9^Research Institute-McGill University Health Centre, Montreal, QC, Canada

## Abstract

**Objectives:**

Acute pain trajectories are associated with long-term outcomes such as persistent pain and functional disability in adults. However, there are limited data on acute postoperative pain trajectories in the pediatric population. The aims of this study were to investigate acute postoperative pain trajectories, their predictors, and their impact on long- term outcomes in adolescents with idiopathic scoliosis.

**Methods:**

We evaluated the preoperative pain intensity, use of analgesics, psychosocial measures and physical functioning of adolescents scheduled to undergo spinal fusion, and their average 6-hour self-reported pain intensity scores for their entire hospital stay. Six months after surgery, baseline variables were reassessed. We used growth mixture modeling to conduct acute postoperative pain trajectory analysis and to identify predictors of pain trajectories. Generalized linear models were conducted to determine whether acute pain trajectories predict long-term outcomes.

**Results:**

One hundred and six patients were included in the best-fitted acute pain trajectory model that included four classes that differed in initial pain intensity and rates of change over time. Preoperative pain catastrophizer status and use of analgesics significantly predicted pain trajectory membership. Furthermore, at the 6-month follow-up, patients experiencing moderate-to-severe pain in the acute postoperative period were more likely to report higher levels of pain severity, use pain medication, and miss a greater number of school/work days due to back pain in the last three months. *Discussion*. Preoperative assessment and analyzing the progression of pain in the acute postoperative period can help identify those at risk of negative long-term outcomes after surgery.

## 1. Introduction

Chronic postsurgical pain is defined as pain that persists for over three months, well after the surgical tissues have healed [1]. Spinal fusion surgery with instrumentation is performed on pediatric patients with adolescent idiopathic scoliosis (AIS), a 3-dimensional deformity of the spine with pronounced single or double curving of the spine [2]. Spinal fusion surgery with instrumentation is an invasive and extensive surgery such that persistent pain is a common postoperative complication [3]. This is highly worrisome as pain can lead to negative consequences such as prolonged emotional distress, long-term pain medication usage, and delayed recovery from surgery [4, 5].

Acute postsurgical pain has been shown to predict chronic pain and opioid use in children and adolescents [6, 7]. Moreover, the persistent nature of postoperative pain may delay rehabilitation [4, 8]. The days following surgery are therefore a critical period where the dynamism of acute pain has an impact on long-term outcomes. Kwan et al. (2017) reported on average pain intensities at 12–24 hour intervals for up to 2 weeks after surgery in a cohort of AIS patients [9]. While pain was considered in relation to time, enormous variability existed in the outcomes at each time point, suggesting heterogeneity of the study population and the presence of multiple subgroups.

Given the heterogeneity present in these populations, patient subgroups with unique pain trajectories may be determined using trajectory analysis. These probabilistic latent class models can capture the progressive change of pain over time [10] that is unique to specific subgroups of individuals. In adults after total hip arthroplasty, Pagé et al. (2016) identified 4 pain trajectories in the acute postoperative period [11]. Importantly, preoperative variables such as pain and anxiety predicted pain trajectory membership. Pain trajectories were in turn associated with long-term outcomes such as pain and functional disability.

Recently, our group conducted acute postoperative opioid consumptions trajectories and their impact on long-term outcomes in a large pediatric cohort of patients undergoing spine surgery [12]. Our findings revealed that patients experiencing mild pain in the acute postoperative period were low-opioid consumers, while those experiencing moderate pain were high-opioid consumers. In addition, the trajectory analysis showed that despite high-opioid consumption, a specific group of adolescents experienced moderate pain that continued to increase up until their hospital discharge (5 days after surgery). In these patients, the analgesic effect of opioids may not have been sufficient enough to provide adequate pain control, thus confirming the need for more personalized pain management. Therefore, the objective of this study was to investigate further this cohort of adolescent patients undergoing spinal fusion surgery by conducting pain trajectory analyses to evaluate if specific acute postoperative pain experiences exist in these patients, to assess the impact of unique acute postoperative pain experiences on long-term outcomes, and to identify predictors of trajectory membership to different postoperative pain experiences.

## 2. Materials and Methods

### 2.1. Study Design

The analyses conducted were part of a larger study approved by the Institutional Review Board of McGill University (A08-M71-17B). Adolescents between 10 and 18 years old with idiopathic scoliosis and scheduled to undergo posterior spinal fusion surgery were prospectively recruited and consented at the Shriners Hospitals for Children-Canada between 2013 and 2018. Exclusion criteria included children unable to speak, write, or read English or French, children diagnosed with developmental delay, and children with major chronic medical conditions. Study variables were assessed at baseline (7–10 days before surgery), at postoperative hours 1 to 120 from the moment the patient leaves the operating room (averaged every 6 hours), and at follow-up 6 months after surgery.

### 2.2. Questionnaires

At baseline, patients completed the Pain Catastrophizing Scale-child (PCS-c) questionnaire to assess their mental state in relation to actual and/or anticipated pain. Patients who received a total score of 30 or greater out of 52 on the PCS-c questionnaire were considered pain catastrophizers [13]. Preoperatively, patients completed the Scoliosis Research Society-30 (SRS-30) questionnaire which has been validated to assess the quality of life and outcomes for individuals with scoliosis scheduled to undergo spinal surgery [14]. Given that pain is the primary outcome variable, we evaluated specific questions separately. These questions included (1) pain experienced in the past 6 months, (2) pain experienced in the last month, (3) pain experienced at rest, (4) current level of activity, (5) sick days from work/school over the past 3 months due to back pain, and (6) medication usage for pain from the following options: none, nonopioids (e.g., Tylenol), or opioids (e.g., Dilaudid). The same SRS-30 variables were reassessed at the patients' 6-month follow-up appointment, instead of a numerical rating scale (NRS) for pain because chronic pain is defined as persistent and/or recurrent pain lasting at least 3 months or longer [1], and there is a lack of evidence for a recommended self-report measure of chronic pain in children and adolescents [15]. The separate questions in the SRS-30 capture the experience of the pain within the last 6 months after their surgery.

### 2.3. Perioperative Anesthesia and Analgesia Care

Perioperative anesthesia was standardized for the study. Intraoperatively, patients received intravenous (IV) propofol, remifentanil/sufentanil/fentanyl, ketamine, and dexamethasone. After induction, patients received an intrathecal injection of morphine (5 *μ*g/kg). Postoperatively, all patients received IV patient-controlled analgesia (PCA) (1 : 1) morphine/ketamine of 20 mcg/kg bolus on demand with a 6-minute lockout interval and a maximum dose per hour of 0,1–0,4 mg/kg/h available upon arrival at the postanesthesia care unit (PACU) till the morning of postoperative day three. Throughout the acute postoperative period, acetaminophen and ketorolac were available on a scheduled and PRN basis. Furthermore, opiates were available after PCA on a scheduled and PRN basis.

### 2.4. Acute Postoperative Pain Assessment

Self-reported pain intensity was assessed by a bedside nurse during the in-hospital period using the NRS 0–10, where 0 indicates no pain, 1–3 indicates mild pain, 4–6 indicates moderate pain, >7 indicates severe pain, and 10 indicates the worst pain imaginable. The NRS has been validated in the pediatric population and is strongly recommended as a self-report measure for acute pain intensity for children and adolescents between 6 and 18 years old [15, 16]. Average self-reported pain intensity was extracted every hour during the acute postoperative period from the patients' electronic medical charts and average postoperative 6-hour pain intensities were calculated for pain trajectory analysis.

### 2.5. Statistical Analysis

Growth mixture modeling (GMM) was used to perform acute postoperative pain trajectory analyses similar to previous studies [11, 12, 17]. GMM considers interindividual variability in intraindividual patterns of longitudinal data to identify and model trajectories of unique subgroups within the sample despite possible missing data [10]. Average 6-hour pain intensities were used as the basis of the analyses. Six linear and six linear + quadratic trajectory models were tested using the heterogeneous linear mixed effects (hlme) function of the latent class mixed model (lcmm) package in R version 3.2.1. Selection of the best trajectory model was based on multiple criteria: low values for Akaike Information Criterion (AIC) and Bayesian Information Criterion (BIC) indicating better fit, high entropy (>80%) reflecting high confidence of group membership, a minimum trajectory class size of 5% of total study population, and parsimony [10, 11, 17]. After the best trajectory model was selected, using the Wald test, pre- and intraoperative factors known to play a predictive role in the acute postoperative pain experience (age, sex, pain medication use, pain severity, pain catastrophizer status, functional activity, largest Cobb angle, number of vertebrae fused, surgery length, blood loss, and intraoperative anesthetic doses) [18, 19] were individually tested as predictors of trajectory membership in the model. Significant variables (*p* < 0.05) were included in the final model. The required minimum sample size of 100 for GMM was based on the theoretical foundations of the study, characteristics of the data, measurement reliability, and group differences [10, 20].

With R version 3.2.1, chi-square test or Kruskal–Wallis H test followed by appropriate post hoc tests was used accordingly to investigate significant differences for reported pain intensity between postoperative days and for perioperative variables between pain trajectory membership. Generalized linear models were conducted to evaluate whether the pain trajectories predict long-term outcomes.

## 3. Results

### 3.1. Study Population

**Figure 1:**
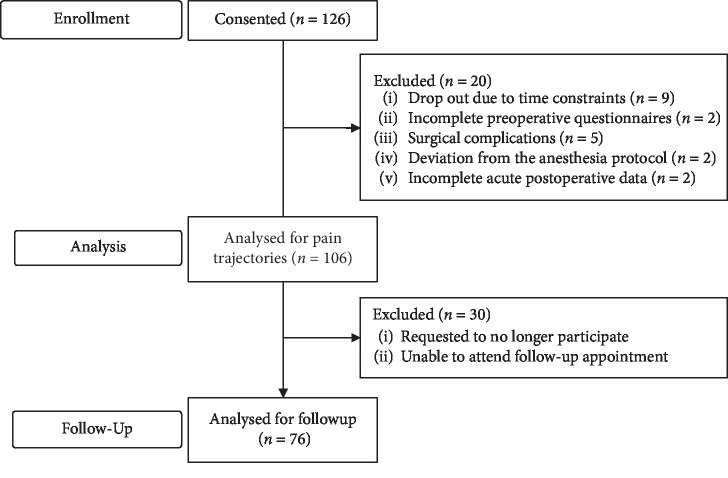
Study flowchart of cohort for the analysis.

One hundred and twenty-six patients consented to participate in the study. However, as depicted in Figure 1, the study population consisted of 106 patients with a mean age of 15.4 ± 2.0 years. Twenty-three patients (25%) were considered to be pain catastrophizers based on their total score on the PSC-c. Within the last 6 months prior to surgery, 9% of the patients reported no pain, 32% experienced mild pain, and 59% experienced moderate-to-severe pain. Additional descriptive statistics are presented in Table 1.

**Figure 2:**
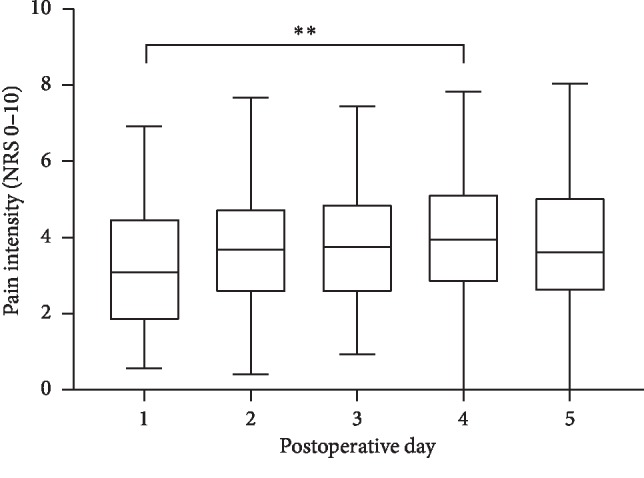
Acute postoperative median pain intensity of patients (*n* = 106). Data are presented as median (middle line), interquartile range (box), and range (whiskers). NRS: numerical rating scale.

The study cohort experienced constant pain for the duration of the acute postoperative period (Figure 2). A significant increase in average pain intensity in our study cohort was only observed between postoperative days 1 and 4 (*p*=0.0046).

### 3.2. Pain Trajectories

**Figure 3:**
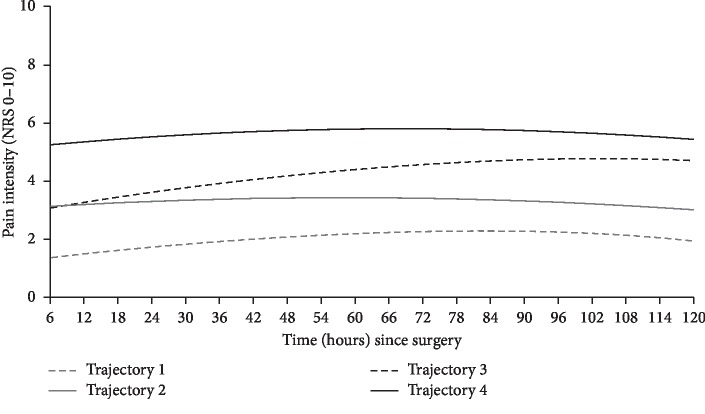
Acute postoperative pain trajectories: trajectory 1 (*n* = 26), trajectory 2 (*n* = 29), trajectory 3 (*n* = 26), and trajectory 4 (*n* = 25). NRS: numerical rating scale.

Goodness-of-fit indices for the twelve tested trajectory models are presented in Table 2. The simplest model with the best fit (AIC = 6959.67; BIC = 7002.28) contained 4 trajectories, a quadratic term, entropies of >0.8 for 92% of patients, and a smallest class size of 24% of the total study population. Pain trajectory 1 (*n* = 26) and pain trajectory 2 (*n* = 29) were characterized by patients who reported mild and mild-to-moderate pain immediately following surgery, respectively, that remained relatively constant throughout the acute postoperative period. Patients in trajectory 3 (*n* = 26) also reported mild-to-moderate pain immediately following surgery but increased steadily to moderate pain by postoperative day five. Pain trajectory 4 (*n* = 25) consisted of patients who reported moderate pain immediately following surgery that remained relatively constant throughout the acute postoperative period. Baseline pain medication use and baseline pain catastrophizing status were significant predictors of trajectory membership in the simplest model and were included in the final model (AIC = 6957.64; BIC = 7016.24) presented in Figure 3. The logarithmic odds that patients were taking pain medication prior to surgery and a member of trajectory 1 instead of trajectory 4 was −1.80 ± 0.86 (Wald *χ*^2^ = −2.1; *p*=0.036). Furthermore, the logarithmic odds that patients are pain catastrophizers and a member of trajectory 1 instead of trajectory 4 was −1.90 ± 0.82 (Wald *χ*^2^ = −2.3; *p*=0.022). No significant differences were observed between pain trajectories regarding continuous preoperative and intraoperative variables. A significant association was observed between pain trajectory membership and baseline pain catastrophizing status (*χ*^2^ = 8.3; *p*=0.04), pain severity in the last six months before the surgery (*χ*^2^ = 21.8; *p*=0.001), or baseline pain medication use (*χ*^2^ = 9.7; *p*=0.02). The intercepts, linear, and quadratic slopes of each trajectory and the predicted and raw pain intensity values of each acute postoperative day are presented in Table 3. Results from the univariate ANOVA of the observed means for each trajectory show that the mean 24-hour pain intensity of each trajectory significantly differed from each other, except between trajectory 2 and trajectory 3 on postoperative day 1 (Table 3).

### 3.3. Prediction of Long-Term Outcomes

Out of the 106 patients, 30 patients were loss to follow-up resulting in the 6-month outcome variables of 76 patients being analyzed. Long-term outcomes after surgery according to pain trajectory can be found in Table 4. A significant association was observed between pain trajectory membership and the pain severity within the last six months postoperatively (*χ*^2^ = 15.8; *p*=0.02), the pain severity within the last month from their follow-up appointment (*χ*^2^ = 14.7; *p*=0.02), the presence of back pain at rest (*χ*^2^ = 11.9; *p*=0.008), and the number of missed days due to back pain in the last three months (*χ*^2^ = 14.6; *p*=0.02).

The results from the generalized linear models are presented in Table 5. At the patient's 6-month follow-up, the results display that acute postoperative pain trajectories significantly predicted postoperative 6-month pain experience, the pain experience in the last month, whether they experience back pain at rest, the number of missed school/work days due to back pain in the last three months, and pain medication use (Table 5). Patients experiencing moderate pain in the acute postoperative period were more likely to report higher levels of pain severity, use pain medication, and miss a greater number of school/work days due to back pain in the last three months at their 6-month follow-up appointment.

## 4. Discussion

Four unique acute pain trajectories were identified that differed in their initial pain intensity and rate of change over time. A moderate-to-severe pain intensity throughout the acute postoperative period predicted negative long-term outcomes. Self-reported pain catastrophizing status and the use of pain medication before surgery predicted higher acute postoperative pain trajectory membership.

In comparison to traditional single and/or mean measures of pain, examining pain in relation to time by trajectory analysis may offer more insight on the impact of different acute postoperative experiences on long-term outcomes [6]. Four unique acute pain trajectories were identified. Visually, patients in trajectories 1, 2, and 4 reported constant pain across the acute postoperative period, while patients in trajectory 3 reported pain that increased with time. This is one of the few studies to conduct probabilistic latent class models which capture the progressive change of pain over time in the acute postoperative period in the pediatric population. In accordance with adult findings following total hip arthroplasty, Pagé et al. (2016) also identified 4 acute postoperative pain trajectories. Interestingly, their study revealed 3 trajectories whose members reported pain that decreased as time progressed and one trajectory whose members reported constant pain [11]. The difference in trajectory patterns between the two studies may be explained by different patient populations, age of study participants, and different surgical scenario [3]. Overall, our findings suggest that pediatric patients experience pain that evolves with time in the acute postoperative period. Furthermore, the identification of multiple trajectories illustrates the presence of latent subgroups in the pediatric population with interindividual differences in pain experience after surgery. Knowledge of these differences warrants the need to consider the variability on pain perception and pain profile following specific surgical procedures and to manage acute postoperative pain as a dynamic event [21].

Acute pain trajectories significantly predicted back pain severity experienced 6 months after surgery. In a preliminary study, our group demonstrated that pain intensity on postoperative days 1 and 2 after a spinal fusion surgery was predictive of pain intensity six weeks after surgery [22]. Although interesting, the follow-up period was too short to make conclusions regarding chronic postsurgical pain. Other studies have shown that modeling acute pain trajectories can give insight on pain chronification. Chidambaran et al. (2017) reported that AIS patients who reported chronic pain 3 months and persistent pain one year after spinal fusion surgery had higher pain trends in the acute postoperative period [23]. Therefore, the acute postoperative period may be a crucial point of intervention to prevent long-term postsurgical pain in AIS patients. Interestingly, acute pain trajectory membership predicted the number of missed school/work days due to back pain, but not the current level of activity at the patients' 6-month follow-up appointment. It is plausible that while acute postoperative pain does not predict long-term physical functioning, it may predict the effect of pain on daily functioning/quality of life. Acute pain trajectory membership also predicted the use of pain medications 6 months after surgery. Consistent with our findings, Fassoulaki et al. (2008) found that, in adult patients after breast cancer surgery, average pain intensity in the first nine postoperative hours significantly predicted use of analgesics 6 months after surgery [24]. However, the latter study used a single average pain intensity measure as opposed to a continuous measure of pain by our study. Therefore, analyzing interindividual variability in the acute postoperative period in pediatrics may give more insight on long-term outcomes and the acute period is a crucial period for intervention to prevent persistent pain and long-term pain medication use. Clinicians should manage pain as a dynamic event in the acute postoperative period and identify patients at risk of negative long-term outcomes to intervene early to prevent chronic postsurgical pain.

Use of pain medications prior to surgery predicted pain trajectory membership. Specifically, patients reporting the highest pain ratings were more likely to have been consuming opioids or nonopioid pain medications at baseline than patients reporting the lowest pain ratings. In an adult patient population undergoing total knee arthroplasty, prolonged (>4 weeks) opioid use before surgery resulted in greater pain in the first 6 days following surgery at rest and while walking than opioid-free patients [25]. Furthermore, a recent meta-analysis on preoperative predictors of poor acute postoperative pain control revealed that preoperative analgesia use was a significant predictor [26]. In our study, pain medications included opioids and nonopioids, where only 5.6% of AIS patients were opioid consumers prior to surgery. We hypothesize that our results may be explained by the phenomenon of opioid-induced hyperalgesia before surgery, where opioid use leads to a decrease in pain threshold and thus a greater experience of pain [27]. The patients who were taking pain medication prior to their surgery may have led to the priming of their nervous system to subsequent pain during the acute postoperative period [28]. However, due to the low proportion of patients consuming opioids preoperatively, it is difficult to correlate our results with this hypothesis. Nevertheless, preoperative intervention to decrease pain medication intake such as emphasizing on nonpharmacological interventions may be suggested instead to reduce the risk of high acute postoperative pain.

Psychological factors are known to affect an individual's pain experience [29]. Pain catastrophizing status was a predictor of trajectory membership such that patients experiencing moderate-to-severe acute postoperative pain were more likely to be pain catastrophizers than patients experiencing mild acute postoperative pain. Although Ferland et al. (2017) could not identify a predictive effect of trait anxiety of AIS patients, or their preoperative anxiety state, on postoperative pain intensity [30], Connelly et al. (2014) reported that AIS patients with greater pain coping efficacy before surgery had a more rapid rate in pain intensity improvement [31]. More evidence shows the importance of mental state before surgery as a major predictor of postoperative pain in pediatrics [32, 33]. It was recently discussed that pain catastrophizing can have an impact on a patient's postoperative pain management through their use of patient-controlled analgesia (PCA) [34]. The acute postoperative period is an important time period where the patient may feel like they are not in control when in pain. LaMontagne et al. (2003) investigated the effect of cognitive-behavioral intervention on adolescents' pain following spinal fusion surgery. In their study, they observed that videotape intervention combining information of the surgical procedure, the sensations felt after surgery, and information on coping behaviors led to less postoperative pain in adolescents with high preoperative anxiety [35]. Overall, our results suggest that it is primordial in pediatrics to identify before their surgery date pain catastrophizers who are considered to possess maladaptive coping behaviors [29] and intervene via pain counselling/education to reduce the risk of high postoperative pain intensity.

A limitation of the present study was that only the patient's psychological factors were evaluated in isolation to the parental ones. Studies have shown that parental psychological factors also play an important role in their children's response to pain [36, 37]. Rabbitts et al. (2014; 2015) observed that parental pain catastrophizing had a significant impact on pain trajectory membership, such that higher parental pain catastrophizing led to greater pain intensity in the acute postoperative period and late recovery after surgery [38, 39]. Another limitation of this study may be the process in which data were collected as the pain ratings found in the medical charts at an interval of 6 hours were averaged. Trajectories 1–2 and trajectories 3–4 are clinically similar representing mild and moderate pain, respectively, in the acute postoperative period. This may be due to the variability in pain response in the acute postoperative period within each 6-hour interval. Future work evaluating the dynamism of pain intensity in the acute postoperative period with different time intervals should be conducted. Another limitation was the small size of the trajectory groups and the number of patients lost to follow-up at 6 months after surgery. This limitation may have led some pre- and/or intraoperative factors to not have a significant predictive role in trajectory analyses and decreases the predictive effect of acute postoperative pain trajectories on long-term outcomes. Furthermore, increasing the follow-up period to one or two years would be ideal to determine whether the acute postoperative period predicts persistent postsurgical pain in pediatrics similarly to previous studies [3, 23, 40]. Increasing the follow-up period is important in our cohort of patients, because pain may only become present 1 year after spinal fusion surgery in AIS patients [41]. Addressing this issue is important and future work should be conducted with additional patients to improve the strength of statistical analysis of long-term outcomes.

## 5. Conclusions

In conclusion, in this study of AIS patients, four acute postoperative pain trajectories were identified. Pain catastrophizer status and pain medication use before surgery are significant predictors of acute postoperative pain trajectories. In turn, pain experience in the acute postoperative period has an impact on patients' postoperative 6-month pain experience, number of missed school/work days due to back pain in the last three months, and pain medication use at their 6-month follow-up appointment. Therefore, preoperative assessment of surgical AIS patients and analyzing their progression of pain in the acute postoperative period can help identify who is at risk of negative long-term outcomes after surgery and allow clinicians to intervene early to prevent persistent postsurgical pain and its negative impact on the daily lives of the patients.

## Figures and Tables

**Table 1:** Preoperative and intraoperative variables of study patients (*n* = 106).

Preoperative and intraoperative variables	Total patient sample (*n* = 106)	Pain trajectory			
		1 (*n* = 26)	2 (*n* = 29)	3 (*n* = 26)	4 (*n* = 25)
Demographics					
Age, years	15.4 (2.0)	15.7 (2.0)	15.5 (2.1)	14.9 (1.6)	15.4 (2.2)
Sex, *n* (%)					
Male	25 (23.6)	8 (30.8)	7 (24.1)	8 (30.8)	2 (8.0)
Female	81 (76.4)	18 (69.2)	22 (75.9)	18 (69.2)	23 (92.0)

Largest cobb angle, °	56.8 (12.6)	54.2 (14.3)	55.0 (13.1)	60.7 (11.5)	57.6 (10.6)
Pain catastrophizing status					
Total PCS-c score	22.9 (9.8)				
^#^Pain catastrophizers, *n* (%)					
No	80 (75.5)	24 (92.3)	20 (69.0)	21 (80.8)	15 (60.0)
Yes	26 (24.5)	2 (7.7)	9 (31.0)	5 (19.2)	10 (40.0)

SRS-30 variables, *n* (%)					
^#^Pain in last 6 months					
None	9 (8.5)	4 (15.4)	0 (0)	2 (7.7)	3 (12.0)
Mild	34 (32.1)	16 (61.5)	8 (27.6)	4 (15.4)	6 (24.0)
Moderate to severe	62 (58.5)	6 (23.1)	21 (72.4)	19 (73.1)	16 (64.0)
Pain in last 1 month					
None	13 (12.3)	7 (26.9)	2 (6.9)	2 (7.7)	2 (8.0)
Mild	29 (27.4)	10 (38.5)	6 (20.7)	6 (23.1)	7 (28.0)
Moderate to severe	64 (60.4)	9 (34.6)	21 (72.4)	18 (69.2)	16 (64.0)
Back pain at rest					
No	17 (16.0)	5 (19.2)	4 (13.8)	6 (23.1)	2 (8.0)
Yes	89 (84.0)	21 (80.8)	25 (86.2)	20 (77)	23 (92.0)
Current level of activity					
Full activities without restriction	45 (42.5)	13 (50)	13 (44.8)	11 (42.3)	8 (32.0)
Light-to-moderate activity	45 (42.5)	9 (34.6)	11 (37.9)	9 (34.6)	16 (64.0)
No activity	16 (15.1)	4 (15.4)	5 (17.2)	6 (23.1)	1 (4.0)
Missed school/work days due to back pain in last 3 months					
0	75 (70.8)	21 (80.8)	22 (75.9)	16 (61.5)	16 (64.0)
1–3	17 (16.0)	3 (11.5)	4 (13.8)	4 (15.4)	6 (25.0)
4 or more	13 (12.3)	2 (7.7)	3 (10.3)	6 (23.1)	2 (8.0)
^#^Medications for pain, *n* (%)					
None	78 (73.6)	24 (92.3)	23 (79.3)	15 (57.7)	16 (64.0)
Yes					
Nonopioids	22 (20.8)	2 (7.7)	4 (13.8)	9 (34.6)	7 (28.0)
Opioids	6 (5.7)	0 (0)	2 (6.9)	2 (7.7)	2 (8.0)

Anesthetic variables					
Remifentanil, mg/kg	45.6 (22.9)	37.0 (23.3)	45.5 (21.2)	51.7 (23.2)	48.5 (22.4)
Sufentanil, mg/kg (*n* = 28)	0.56 (0.52)	0.26 (0.14)	0.67 (0.65)	0.45 (0.37)	0.79 (0.65)
Fentanyl, mg/kg (*n* = 14)	2.30 (0.55)	2.7 (1.3)	2.2 (0.45)	2.5 (0.35)	2.0 (0.38)
Dexamethasone, mg/kg	0.11 (0.04)	0.12 (0.03)	0.10 (0.01)	0.12 (0.04)	0.12 (0.04)
Ketamine, mg/kg	0.70 (0.57)	0.70 (0.45)	0.73 (0.52)	0.74 (0.50)	0.72 (0.81)
Spinal morphine, mg/kg	5.40 (2.97)	5.28 (2.77)	5.96 (3.92)	5.11 (1.62)	5.19 (3.05)
Total intraoperative opioids (equivalents of morphine), mg/kg	36.1 (17.0)	29.5 (17.7)	36.3 (15.8)	40.7 (17.1)	38.3 (16.6)

Surgical variables					
Surgery length, minutes	264.6 (76.4)	254.7 (71.6)	260.3 (75.8)	277.3 (76.5)	266.5 (84.1)
Blood loss, mL	761.1 (441.2)	769.5 (437.4)	790.5 (509.1)	721.7 (300.0)	759.5 (503.3)
Number of fused vertebrae	10.6 (2.6)	10.2 (2.8)	10.0 (2.9)	11.5 (1.6)	10.9 (2.5)

Data are presented as mean (SD), unless otherwise specified. PCSc: Pain Catastrophizing Scalechild questionnaire, SRS-30: Scoliosis Research Society questionnaire: version 30. ^#^Significant association observed between the variable and pain trajectory membership.

**Table 2:** Goodness of fit indices for the twelve tested trajectory models.

Number of trajectories	Linear			Linear + quadratic		
	AIC	BIC	SC (%)	AIC	BIC	SC (%)
1	8170.13	8178.12	100	8159.95	8170.6	100
2	7346.11	7362.09	43.4	7328.15	7349.46	43.4
3	7057.98	7081.95	26.4	7035.83	7067.79	26.4
4	6982.58	7014.54	23.6	6959.67^*∗*^	7002.28^*∗*^	23.6^*∗*^
5	6933.27	6973.22	11.3	6915.3	6968.57	11.3
6	6908.45	6956.39	7.5	6879.58	6943.5	9.4

^*∗*^The model with the best fit. AIC: Akaike Information Criterion. BIC: Bayesian Information Criterion. SC: smallest class size.

**Table 3:** Description of final pain trajectory model, predicted and raw acute postoperative pain intensity values.

Pain trajectory	Slopes				Predicted values (NRS 0–10)					Raw values (NRS 0–10), mean (SD)				
	*n*	Intercept	Linear	Quadratic	POD1	POD2	POD3	POD4	POD5	POD1	POD2	POD3	POD4	POD45
1	26	1.21162	0.0277	−0.00018	1.77	2.13	2.28	2.21	1.95	1.57 (0.66)^a,b,c^	1.88 (0.71)^a,b,c^	1.94 (0.49)^a,b,c^	2.40 (1.18)^a,b,c^	1.91 (0.72)^a,b,c^
2	29	3.06402	0.0128	−0.00011	3.31	3.42	3.42	3.28	3.02	3.10 (1.03)^a,e^	3.44 (0.67)^a,d,e^	3.33 (0.55)^a,d,e^	3.32 (0.73)^a,d,e^	3.09 (0.66)^a,d,e^
3	26	2.85109	0.0348	−0.00016	3.59	4.15	4.52	4.71	4.72	3.21 (1.06)^b,f^	4.07 (0.83)^b,d,f^	4.25 (0.64)^b,d,f^	4.88 (0.85)^b,d,f^	4.75 (1.06)^b,d,f^
4	25	5.15001	0.0192	−0.00014	5.53	5.75	5.81	5.71	5.44	5.40 (0.90)^c,e,f^	5.63 (0.85)^c,e,f^	5.74 (0.69)^c,e,f^	5.82 (1.26)^c,e,f^	5.65 (1.18)^c,e,f^

										*F* = 73.2	*F* = 104.9	*F* = 182.3	*F* = 58.4	*F* = 84.2

										*p* < 0.001	*p* < 0.001	*p* < 0.001	*p* < 0.001	*p* < 0.001

**Table 4:** Long-term outcomes according to pain trajectory membership.

Long-term outcomes	Pain trajectory			
	1 (*n* = 19)	2 (*n* = 22)	3 (*n* = 17)	4 (*n* = 18)
6-month SRS-30 variables, *n* (%)				
^#^Pain in last 6 months				
None	9 (47)	2 (9)	2 (12)	2 (11)
Mild	8 (42)	8 (36)	7 (41)	7 (39)
Moderate to severe	2 (11)	12 (55)	8 (47)	9 (50)
^#^Pain in last 1 month				
None	11 (61)	7 (32)	3 (18)	3 (17)
Mild	8 (44)	8 (36)	9 (53)	7 (39)
Moderate to severe	0 (0)	7 (32)	5 (29)	8 (44)
^#^Back pain at rest				
No	11 (58)	9 (41)	5 (29)	1 (6)
Yes	8 (42)	13 (59)	12 (71)	17 (94)
Current level of activity				
Full activities without restriction	3 (16)	1 (5)	2 (12)	1 (6)
Light-to-moderate activity	15 (79)	19 (86)	13 (76)	15 (83)
No activity	1 (5)	2 (9)	2 (12)	2 (11)
^#^Missed school/work days due to back pain in last 3 months				
0	16 (84)	19 (90)	11 (65)	8 (45)
1–3	3 (16)	0 (0)	3 (17.5)	6 (33)
4 or more	0 (0)	2 (10)	3 (17.5)	4 (22)
Medications for pain				
None	16 (84)	15 (68)	11 (65)	9 (50)
Yes				
Nonopioids	3 (16)	6 (27)	6 (35)	6 (33)
Opioids	0 (0)	1 (5)	0 (0)	3 (17)

SRS-30, Scoliosis Research Society questionnaire, version 30. ^#^Significant association observed between the variable and pain trajectory membership.

**Table 5:** Prediction of long-term outcomes.

Long-term outcomes independent variable	Pain in last 6 months	Pain in last month	Back pain at rest	Current level of activity	Missed school/work days due to back pain in last 3 months	Pain medication use
Pain trajectory 1	*b* ± SE (*p* value)	*b* ± SE (*p* value	*b* ± SE (*p* value	*b* ± SE (*p* value)	*b* ± SE (*p* value)	*b* ± SE (*p* value)
Pain trajectory 2	2.20 ± 0.87 (0.012)	1.08 ± 0.65 (0.098)	0.69 ± 0.64 (0.28)	1.37 ± 1.20 (0.25)	−0.58 ± 0.97 (0.55)	0.92 ± 0.78 (0.24)
Pain trajectory 3	1.91 ± 0.88 (0.030)	1.86 ± 0.79 (0.018)	1.19 ± 0.71 (0.091	0.34 ± 0.98 (0.73)	1.07 ± 0.81 (0.19)	1.07 ± 0.81 (0.19)
Pain trajectory 4	1.97 ± 0.88 (0.025)	1.93 ± 0.78 (0.014)	3.15 ± 1.13 (0.0053)	1.16 ± 1.21 (0.34)	1.90 ± 0.79 (0.016)	1.67 ± 0.79 (0.033)

## Data Availability

The data used to support the findings of this study are available from the corresponding author upon request.
